# Indoor Environmental Exposures for Children with Asthma Enrolled in the HEAL Study, Post-Katrina New Orleans

**DOI:** 10.1289/ehp.1104840

**Published:** 2012-08-15

**Authors:** L. Faye Grimsley, Patricia C. Chulada, Suzanne Kennedy, LuAnn White, Jeremy Wildfire, Richard D. Cohn, Herman Mitchell, Eleanor Thornton, Jane El-Dahr, Mosanda M. Mvula, Yvonne Sterling, William J. Martin, Kevin U. Stephens, Maureen Lichtveld

**Affiliations:** 1 School of Public Health and Tropical Medicine, Tulane University, New Orleans, Louisiana, USA; 2 Clinical Research Program, National Institute of Environmental Health Sciences, National Institutes of Health, Department of Health and Human Services, Research Triangle Park, North Carolina, USA; 3 Rho Federal Systems Division, Inc., Chapel Hill, North Carolina, USA; 4 SRA International, Inc., Durham, North Carolina, USA; 5 Visionary Consulting Partners, LLC, Fairfax Station, Virginia, USA; 6 School of Medicine, Tulane University, New Orleans, Louisiana, USA; 7 New Orleans Health Department, New Orleans, Louisiana, USA; 8 Health Sciences Center School of Nursing, Louisiana State University, New Orleans, Louisiana, USA; 9 National Institute of Child Health and Development, National Institutes of Health, Department of Health and Human Services, Bethesda, Maryland, USA

**Keywords:** allergens, asthma, endotoxin, environmental remediation, glucan, Hurricane Katrina, mold

## Abstract

Background: Rain and flooding from Hurricane Katrina resulted in widespread growth of mold and bacteria and production of allergens in New Orleans, Louisiana, which may have led to increased exposures and morbidity in children with asthma.

Objectives: The goal of the Head-off Environmental Asthma in Louisiana (HEAL) study was to characterize post-Katrina exposures to mold and allergens in children with asthma.

Methods: The homes of 182 children with asthma in New Orleans and surrounding parishes were evaluated by visual inspection, temperature and moisture measurements, and air and dust sampling. Air was collected using vacuum-pump spore traps and analyzed for > 30 mold taxa using bright field microscopy. Dust was collected from the children’s beds and bedroom floors and analyzed for mouse (Mus m 1), dust mite (Der p 1), cockroach (Bla g 1), and mold (*Alternaria* mix) allergens using ELISA.

Results: More than half (62%) of the children were living in homes that had been damaged by rain, flooding, or both. Geometric mean indoor and outdoor airborne mold levels were 501 and 3,958 spores/m^3^, respectively. *Alternaria* antigen was detected in dust from 98% of homes, with 58% having concentrations > 10 µg/g. Mus m 1, Der p 1, and Bla g 1 were detected in 60%, 35%, and 20% of homes, respectively, at low mean concentrations.

Conclusions: Except for *Alternaria* antigen in dust, concentrations of airborne mold (ratio of indoor to outdoor mold) and dust allergens in the homes of HEAL children were lower than measurements found in other studies, possibly because of extensive post-Katrina mold remediation and renovations, or because children moved into cleaner homes upon returning to New Orleans.

On 29 August 2005, Hurricane Katrina struck the Gulf Coast, devastating much of the area in its path. The next day, many of the levees in Orleans Parish were breached, and 80% of the parish flooded; water levels rose to 20 feet in some areas (Kates et al. 2006). In addition, high winds caused roof damage, resulting in further water intrusion and moisture. The wet conditions provided an ideal environment for mold and bacterial growth in the city of New Orleans and surrounding parishes (NOLA). These organisms and their by-products (glucans, endotoxins) and allergens that are associated with moist environments (e.g., dust mite, cockroach) can exacerbate childhood asthma and allergies (Clark et al. 2004; Fisk et al. 2007; Jaakkola et al. 2005; Rosenbaum et al. 2010; Rosenstreich et al. 1997). However, the levels at which health effects occur and the factors associated with individual susceptibility to these outcomes are not well characterized (Brandt et al. 2006; Horner et al. 2008).

Several months after Katrina, many homes exhibited visible mold growth. In one study, 46% of 112 homes representing a random cross-section of homes in several NOLA parishes had visible mold growth (Ratard et al. 2006). In other studies, indoor airborne mold concentrations were higher than outdoor concentrations (Rao et al. 2007; Solomon et al. 2006). Indoor airborne mold concentrations were especially high in homes that had flooded (Chew et al. 2006), sometimes as much as five times higher than in homes that had not flooded (*p <* 0.05) (Solomon et al. 2006). The high mold concentrations were a concern for children with asthma returning to NOLA because associations between mold and asthma, wheeze, and other respiratory symptoms have been observed in other studies (Clark et al. 2004; Fisk et al. 2007; Jaakkola et al. 2005; Rockwell 2005; Rosenbaum et al. 2010). High concentrations of airborne endotoxin (Rao et al. 2007; Solomon et al. 2006) and glucan (Rao et al. 2007) were also reported in these post-Katrina studies at levels previously associated with respiratory pathology and decreased lung function (Douwes et al. 2002; Reynolds et al. 2001; Rylander et al. 1998).

HEAL was implemented to better understand the risk factors and needs of children with moderate-to-severe asthma in postdisaster settings such as post-Katrina NOLA. Its primary goals were to *a*) characterize post-Katrina environmental exposures; *b*) examine relationships between exposures, allergen sensitivities, and asthma morbidity; and *c*) implement a novel hybrid asthma counselor program that combined case management with tailored guidance for reducing allergens in the home (Chulada et al. 2012). Home environment evaluations were an important component of HEAL. Results from these evaluations were used to characterize post-Katrina environmental exposures in children with asthma as described above, tailor the asthma counselor intervention to the child’s sensitivity and exposure, and assess the effectiveness of the asthma counselor intervention at reducing allergens in the home. In this report, we describe the baseline environmental exposures (airborne mold and dust levels of allergens, endotoxin, and glucan) of HEAL children. These data are important in light of the dynamic nature of the post-Katrina environment, and will be instrumental in further analysis of the relationships between asthma, mold, and allergens in a postdisaster setting.

## Methods

*Participants.* Eligibility criteria, recruitment methods, and characteristics of the 182 HEAL children have been described elsewhere (Chulada et al. 2012). Children were recruited based on having physician-diagnosed, moderate-to-severe asthma, as defined by the 2007 National Asthma Education and Prevention Program (National Heart Lung and Blood Institute 2007). Although more than half of HEAL children (62%) came from water-damaged homes, this was not a recruitment criterion. HEAL was approved by the National Institute of Environmental Health Sciences, Tulane University, and Louisiana State University institutional review boards; written informed consent was provided by each child’s caretaker or legal guardian, and children were asked for written or oral assent, depending on age.

*Home environment evaluations.* The homes of HEAL children were evaluated by teams of two trained environmental technicians. The evaluations lasted about 2.5 hr and consisted of a visual inspection, moisture and temperature measurements, a home survey with caregivers to collect residential and remediation histories, and environmental sample collection.

*Visual inspections*. Technicians followed a standardized protocol for conducting visual inspections that was modeled on the Inner-City Asthma Study (ICAS) (Crain et al. 2002) but modified for HEAL. The homes were inspected to determine the dwelling type and size, collect information on floor coverings and window treatments, and record the presence of dirty or greasy kitchen appliances, dehumidifiers, high-efficiency particulate air (HEPA) filtration units, animals, and odors [e.g., mustiness, tobacco smoke; formaldehyde if a FEMA (Federal Emergency Management Agency) trailer]. Homes were also inspected for signs of mold, moisture, water damage, roof leaks, smoking, open food containers, crumbs, pest infestation, and other sources of allergens and environmental hazards. The technicians focused on the living room, kitchen, child’s sleeping room, and child’s bathroom.

*Moisture and temperature measurements*. Indoor air temperature and relative humidity were measured as an indicator of dampness using a thermohygrometer pen (Fisher Scientific, Pittsburgh, PA). Outdoor temperature and humidity were also measured.

*Home survey*. The home surveys were based on instruments used in ICAS (Crain et al. 2002; Morgan et al. 2004) that were modified for HEAL. In the HEAL version, questions were added regarding the pre- and post-Katrina presence of mold and post-Katrina water damage and roof leaks, renovation (gutting walls, replacing floors and ceilings), and remediation for mold (scrubbing, treating or pressure washing walls or floors, replacing porous furnishings or heating, ventilation, and air conditioning systems).

*Airborne mold sampling procedures*. Indoor airborne mold samples were collected from the children’s bedrooms and living rooms using Zefon Air-O-Cell spore traps and battery-operated Zefon Bio-Pumps (Zefon International Inc., Ocala, FL). The spore trap and pump assembly was attached to a Bio-Pump tripod (Zefon International Inc.) and placed in the center of each room approximately 1.2 m above the floor. Duplicate air samples were collected from each room for 10 min at a flow rate of 15 L air/min. The time was based on Zefon recommendations for indoor sampling where no visible dust is evident. One outdoor airborne mold sample was collected approximately 6 m from the front door of the home using the same technique.

For the baseline dataset, one bedroom air sample and one outdoor air sample were analyzed at Air Quality Sciences (AQS) (Marietta, GA), an AIHA-EMLAP (American Industrial Hygiene Association–Environmental Microbiology Laboratory Accreditation Program) accredited laboratory, using bright field optical microscopy (Zeiss, Melrose, NY). The glass slips contained within the cassettes were removed, affixed onto regular glass microscope slides, and mounted in lactic acid with cotton blue. Particle deposit traces were located at a magnification of 100×. Large spores (≥ 7 μm) present on 100% of the deposit trace were identified and counted at a magnification of 400×. Small spores (< 7 μm) present on 25% of the deposit trace and spore types that were too numerous to count under 400× were identified and counted at a magnification of 1,000×. Spores were categorized by morphological groups following general taxonomic guidelines. The levels of total spores and specific spore types were expressed as spores per cubic meter of air.

*Dust allergen collection and analysis.* Dust was collected from the children’s beds and bedroom floors using Mitest collectors and filters (Indoor Biotechnologies, Charlottesville, VA) that were fitted onto the nozzle of a standard vacuum cleaner (Eureka Mighty Mite canister 3670G; Eureka, Peoria, IL) (Gruchalla et al. 2005). Technicians vacuumed a 1 m × 1 m square area of floor for 5 min. The Mitest collectors and filters were changed and the wand extension cleaned before the beds were vacuumed; dust was collected from the entire bed for 5 min.

Dust samples were analyzed at AQS. Bed dust was sieved using 50-mesh (300-μm openings) screens. An aqueous extract was prepared by adding 2 mL phosphate-buffered saline (PBS)/0.05% Tween 20 to 100 mg of sieved dust. Dust suspensions were mixed on a tube rotator (Multi-purpose rotator; Scientific Labs, Bohemia, NY) for 2 hr at room temperature and then clarified by centrifugation at 4°C, and the supernatants were frozen until use. If the quantity of bed dust was insufficient to make the extract, it was supplemented or replaced with bedroom floor dust.

Supernatants were analyzed for Der p 1 (dust mite), Bla g 1 (cockroach), and Mus m 1 (mouse) allergens using monoclonal antibody-based (or monoclonal/polyclonal for Bla g 1) enzyme-linked immunosorbent assays (ELISA) (Indoor Biotechnologies, Charlottesville, VA). Results were expressed as the mass of allergen per gram of dust, or as units per gram in the case of Bla g 1. Detection limits were 0.2 µg/g for Der p 1, 0.015 µg/g for Mus m 1, and 0.4 U/g for Bla g 1. Dust samples were also assayed for mold (*Alternaria* mix) by inhibition ELISA using polyclonal antibodies raised against a crude extract of *Alternaria alternata* (Greer Laboratories, Inc., Lenoir, NC). This assay had a detection limit of 0.3 µg/g (Salo et al. 2005). Previous cut-offs associated with increased allergic sensitization are used to establish “high” exposure levels for Bla g 1 (Eggleston et al. 1998), Der p 1 (Platts-Mills et al. 1992), and Mus m 1 (Phipatanakul et al. 2000). Although no standard cut-off has been proposed for *Alternaria*, data from the National Survey of Lead and Allergens in Housing showed that only 25% of U.S. homes have *Alternaria* > 10 µg/g (Salo et al. 2005).

*Endotoxin and glucan analyses.* Sieved bedroom dust was assayed for endotoxin and glucan by the laboratory of Peter Thorne, Environmental Health Sciences Research Center, University of Iowa (Blanc et al. 2005). Dust was suspended to concentrations of 50 mg/mL using pyrogen-free water containing 0.05% Tween 20 and shaken for 1 hr at room temperature. Samples were then centrifuged for 20 minutes at 600 × *g* at 4^o^C, and 0.1-mL aliquots of supernatant were removed to pyrogen-free cryovials for analysis.

Endotoxin was analyzed using the Limulus Amebocyte Lysate Kinetic-QCL Test Kit (BioWhittaker, Walkersville, MD). Standards were diluted in clean borosilicate glass tubes (heated > 2 hr at 200^o^C to remove endotoxin). Two-fold serial dilutions were prepared using pyrogen-free water containing 0.05% Tween 20. A 12-point calibration curve ranging from 0.05 to 100 EU endotoxin/mL gave a correlation of 0.997 to 0.998 based on the maximum change of optical density over time.

Glucan was extracted by adding 0.1 mL of 10× PBS to the supernatants from above. These were shaken for 1 hr at room temperature, autoclaved for 1 hr at 120^o^C, shaken for 15 minutes, and centrifuged at 600 × *g* for 20 min at 4^o^C. The concentration of fungal glucan in the eluates was quantified by sandwich ELISA using custom mouse anti-(1□3, 1□6)-β-d-glucan monoclonal antibody as the capture antibody and rabbit anti-scleroglucan polyclonal antibody as the detection antibody (Blanc et al. 2005).

*Statistical methods*. Log-transformations were used for all airborne mold analyses because the values were log-normally distributed. Results are presented as geometric means and standard deviations. Population-level differences between mold levels in flooded and nonflooded homes were compared using *t*-tests, and the relationship between mold level and flooding was examined in subgroups of interest using both between-group (interaction *F*-tests) and within-group (*t*-tests) differences. Unadjusted baseline correlations between exposures and home characteristics were calculated using Spearman correlations. Analyses were conducted using SAS version 9.2 (SAS Institute Inc., Cary, NC) and R version 2.13 (R Project for Statistical Computing, Vienna, Austria).

## Results

*Home surveys and visual inspection.* Baseline home environmental evaluations occurred between 19 and 35 months post-Katrina (March 2007 through July 2008). A total of 182 homes were evaluated; the predominant type of dwelling was a single-family house (62%). Only nine of the dwellings (5%) were FEMA trailers (Table 1). Mold was visually detected in 21 (12%) of the homes, and a musty smell was detected in 7 (4%) of the children’s bedrooms. Thirty-two percent of the homes had pets (dog only, 23%; cat only, 6%; both, 3%). Problems with cockroaches were reported by caregivers in 40% of the homes, but visual signs of cockroaches were seen in only 16%. Average (± SD) temperature and relative humidity in the children’s bedrooms on the day of the baseline home evaluation were 75.2 ± 4.1^o^F and 51.9 ± 8.9%, respectively.

**Table 1:** HEAL baseline characteristics.

	*n* (%) or mean ± SD
Home characteristics (182 enrolled homes)
Katrina-related water damage to current home
Flooding only	43 (24)
Roof leak only	45 (25)
Flooding and roof leak	25 (14)
None	69 (38)
Current housing type
Single-family detached house	112 (62)
Multifamily house (duplex/triplex/row house)	39 (21)
Apartment	15 (8)
FEMA trailer	9 (5)
Other	7 (4)
Visible mold in the homea	21 (12)
Musty smell in child’s bedroom	7 (4)
Pets
Dog	41 (23)
Cat	11 (6)
Both	5 (3)
Evidence of cockroaches in the home
Reported	72 (40)
Observed	29 (16)
Temperature (°F) and relative humidity (%)
Bedroom temperature	75.2 ± 4.1
Outdoor temperature	81.5 ± 11.3
Bedroom relative humidity	51.9 ± 8.9
Outdoor relative humidity	55.1 ± 17.1
Renovation and remediation of flooded homes before baseline
Renovation status of 68 flooded homes at baseline
Completed	41 (60)
Ongoing	18 (26)
Not started	9 (13)
Types of renovation completed (22 homes)b
Gutted walls	17 (77)
Replaced ceilings	16 (73)
Replaced flooring	20 (91)
Mold remediation completed (22 homes)	20 (91)
Types of mold remediation completed (20 homes)
Scrubbed, treated, or pressure-washed walls	5 (25)
Scrubbed, treated, or pressure-washed studs	10 (50)
Scrubbed, treated, or pressure-washed floors	7 (35)
Replaced porous furnishings	20 (100)
Replaced heating/cooling system	15 (75)
Cleaned vents/ducts	14 (70)
aInformation available for 179 homes only. bDetails about the types of renovation and mold remediation that occurred were collected for 22 of 41 homes that had renovations completed before HEAL enrollment; 20 of these 22 homes also completed mold-specific remediation.	

More than half of the participants (63%) were living in homes that had been water-damaged after Katrina (Table 1); 24% had flood damage only, 25% had roof leaks only, and 14% had both. Of the 68 homes that flooded, 41 had completed renovations and mold remediation before HEAL baseline evaluations were conducted, and details about the steps taken were available for 22 of the 41 homes (Table 1).

*Dust allergens. Alternaria* antigen was detected in dust from 98% of the bedrooms (Table 2) with a median concentration of 13.7 µg/g. The majority of the bedrooms (58%) had *Alternaria* antigen concentrations ≥ 10 µg/g. Mus m 1 (mouse) allergen was the next most prevalent allergen (detected in 60% of bedrooms), with high concentrations (≥ 1.6 µg/g) detected in 3% of the bedrooms. Bla g 1 (cockroach) allergen was detected in 20% of bedrooms, with 4% having high concentrations (≥ 2 U/g). Der p 1 (dust mite) allergen was detected in 35% of bedrooms, with 9% having high concentrations (≥ 2 µg/g).

**Table 2:** HEAL baseline allergen levels and mold sampling results.

	Geometric mean (GSE)	*n* (%) detectable^a^	*n* (%) high^b^
Bedroom dust–allergen levels (181 homes)
Alternaria (µg/g)	11.3 (1.1)	178 (98)	105 (58)
Cockroach [Bla g 1 (U/g)]	0.30 (0.02)	37 (20)	8 (4)
Dust mite [Der p 1 (µg/g)]	0.22 (0.02)	63 (35)	16 (9)
Mouse [Mus m 1 (µg/g)]	0.04 (0.01)	108 (60)	5 (3)
Bedroom dust–microbial components (181 homes)
β-glucan (µg/g)	0.50 (0.06)
Endotoxin (EU/mg)	11.4 (1.5)
Mold air sampling (182 homes)						
Indoor total (spores/m3)	501 (48)
Outdoor total (spores/m3)	3,958 (467)
aDetection limits were 0.4 U/g for Bla g 1, 0.2 µg/g for Der p 1, 0.015 µg/g for Mus m 1, and 0.3 µg/g for Alternaria. bHigh levels were above 2 U/g for Bla g 1, 2 µg/g for Der p 1, 1.6 µg/g for Mus m 1, and 10 µg/g Alternaria.						

*Airborne mold.* Geometric mean airborne mold levels were found to be lower indoors than outdoors (501 spores/m^3^ indoors and 3,859 spores/m^3^ outdoors) (Table 2). There was no obvious time trend in indoor or outdoor airborne mold levels during HEAL [see Supplemental Material, Figure S1 (http://dx.doi.org/10.1289/ehp.1104840)]. A small increase in indoor and outdoor levels was observed in the fall (September, October, and November) of 2007, but differences between fall and non-fall months were not significant (*p =* 0.25 for indoor; *p =* 0.89 for outdoor).

Mold spore taxa in indoor and outdoor air, ranked by prevalence, are presented in Table 3. Molds commonly found in leaf surface/outdoor taxa (e.g., Basidiospores, 98%; *Cladosporium,* 97%; Ascospores, 92%) were detected most frequently indoor and reflected what was detected outdoors and molds associated with severe water damage (e.g., *Ulocladium*, 18%; *Chaeromium*, 15%; *Stachybotrys*, 3%) were not as commonly detected indoors and also reflected what was detected outdoors.

**Table 3:** Levels of airborne mold in HEAL homes at baseline (*n* = 182 homes).

Rank	Indoor molds		Geometric mean (GSE)	Outdoor molds		Geometric mean (GSD)
	Mold type	*n* (%) detectable		Mold type	*n* (%) detectable	
1st	Basidiospores	179 (98)	143 (17.8)	Basidiospores	181 (99)	1,311 (202.7)
2nd	Cladosporium	176 (97)	64 (7.5)	Cladosporium	181 (99)	481 (60.4)
3rd	Ascospores	167 (92)	32 (3.6)	Ascospores	181 (99)	251(29.4)
4th	Penicillium–Aspergillus	142 (78)	17 (2.5)	Penicillium–Aspergillus	133 (73)	19 (2.9)
5th	Curvularia	140 (77)	13 (1.6)	Ganoderma	134 (74)	14 (2.1)
6th	Unidentified	111 (61)	5 (0.6)	Curvularia	122 (67)	11 (1.7)
7th	Bipolaris	106 (58)	5 (0.6)	Unidentified	117 (64)	8 (1.2)
8th	Pithomyces	100 (55)	5 (0.6)	Cercospora	114 (63)	7 (0.9)
9th	Alternaria	72 (40)	3 (0.3)	Alternaria	118 (65)	7 (0.8)
10th	Ganoderma	60 (33)	2 (0.2)	Bipolaris	108 (59)	5 (0.7)
11th	Epicoccum	51 (28)	2 (0.2)	Epicoccum	82 (45)	3 (0.4)
12th	Ulocladium	32 (18)	2 (0.1)	Pithomyces	67 (37)	3 (0.3)
13th	Chaetomium	27 (15)	1 (0.1)	Pestalotia	42 (23)	2 (0.1)
14th	Arthrinium	17 (9)	1 (0.1)	Arthrinium	13 (7)	1 (0.08)
15th	Cercospora	23 (13)	1 (0.1)	Chaetomium	17 (9)	1 (0.09)
16th	Pestalotia	20 (11)	1 (0.1)	Stachybotrys	8 (4)	1 (0.06)
17th	Stemphylium	8 (4)	1 (0.04)	Ulocladium	9 (5)	1 (0.04)
18th	Stachybotrys	6 (3)	1 (0.03)	Stemphylium	9 (5)	1 (0.04)
19th	Trichoderma	1 (1)	1 (0.03)	Rust	5 (3)	1 (0.03)
20th	Rust	2 (1)	1 (0.02)	Trichoderma	1 (1)	1 (0.02)
21st	Other	0 (0)	0 (0)	Other	0 (0)	0 (0)

*Flooded versus nonflooded homes.* Flooded homes (*n* = 68) had marginally higher levels of airborne mold than non-flooded homes (*n* = 114) [627 vs. 438 spores/m^3^ for indoor, *p =* 0.07 (Table 4); 4,954 vs. 3,462 for outdoor, *p* = 0.15]. Flooded homes also had higher levels of indoor airborne mold compared with nonflooded homes when children lived in the same homes during Katrina and at HEAL baseline (*p =* 0.02). Homes that were flooded and also had roof leaks had higher mold levels (742 spores/m^3^) than homes that had roof leaks only or that did not flood or have a roof leak (438 spores/m^3^ for both, *p =* 0.15 and 0.05, respectively). Mold spore levels were consistently higher in flooded versus nonflooded homes, though the differences were not statistically significant. As seen with the total population, the geometric mean airborne mold levels were found to be lower indoors than outdoors for both flooded and nonflooded homes. Also as seen with the total population, molds commonly found in leaf surface/outdoor taxa (e.g., Basidiospores, *Cladosporium*, Ascospores) were detected most frequently indoors and out, whereas molds associated with water damage (e.g., *Ulocladium*, *Chaeromium*, *Stachybotrys*) were not commonly found indoors and outdoors and were distributed similarly in flooded and nonflooded homes [see Supplemental Material, Tables S1 and S2 (http://dx.doi.org/10.1289/ehp.1104840)]. Allergen concentrations measured in bedroom dust (*Alternaria* antigen, Der p 1, Mus m 1, and Bla g 1) did not differ between flooded and nonflooded homes (all *p >* 0.30, data not shown).

**Table 4:** Relationships between home flooding and levels of indoor airborne mold.

	Flooded		Not flooded		*p*-Value^a^
	*n*	Level	*n*	Level	
All participants	68	627	114	438	0.07
Current home is same home child lived in during Katrina
Yes	36	679	56	359	0.02
No	32	573	58	531	0.79
Current home, renovated after Katrina
Yes	41	587	44	445	0.29
No	27	693	70	434	0.14
Current home, had roof leak during Katrina
Yes	25	742	45	438	0.15
No	43	568	69	438	0.27
Current home residence typeb
Single-family	49	613	63	375	0.05
Multifamily	13	799	26	557	0.40
Apartment	4	380	11	365	0.96
FEMA trailer	0	—	9	711	—
Values are geometric means in spores per cubic meter. ap-Values test for differences between homes that were flooded and not flooded. Interaction p-values between the flood status and the given strata were not statistically significant (all p > 0.10). bSingle-family refers to a detached house or non-FEMA trailer; multifamily refers to a duplex, triplex, or row house.										

The 41 homes that flooded but were completely renovated before the baseline evaluation had indoor mold levels of 587 spores/m^3^, compared with 693 spores/m^3^ for the 27 homes that flooded but were not completely renovated by baseline (*p =* 0.63, Table 3). Of the 27 flooded homes that had not been completely renovated by baseline, 14 had renovations that were completed during the course of the study. The changes in airborne mold levels from baseline to 12 months in these 14 homes (406 to 235 spores/m^3^) were not significantly different than the changes observed in the 6 homes that remained unrenovated or had incomplete renovations (1,312 to 327 spores/m^3^, comparison *p =* 0.35). Seven of the 27 flooded homes did not have 12-month evaluations.

*Correlations between exposures and baseline home conditions*. A small correlation was observed between indoor airborne mold levels and bedroom relative humidity (*r* = 0.22, *p =* 0.003) (Table 5). A correlation was observed between β-d-glucan and *Alternaria* antigen concentrations (*r* = 0.26, *p =* 0.006) (Table 5). An inverse correlation was observed between airborne bedroom mold and *Alternaria* allergen levels in bedroom dust (*r* = –0.16, *p =* 0.04).

**Table 5:** Relationships between home characteristics and mold, β-d-glucan, and endotoxin levels.

Correlations (*r*-values)	Indoor air mold (*n* = 182)	Outdoor air mold (*n* = 182)	*Alternaria* (dust) (*n* = 181)	β-d-glucan (*n* = 109)	Endotoxin (*n* = 140)
Indoor mold	—
Outdoor mold	0.40**	—
Alternaria (bedroom dust)	–0.16**	0.00	—
β-d-glucan	0.01	–0.09	0.26**	—
Endotoxin	0.09	0.09	0.04	0.34*	—
Flood level (feet)	–0.14	–0.03	0.06	0.18	–0.04
Bedroom relative humidity	0.22**	0.19**	–0.09	0.02	–0.03
Outdoor relative humidity	0.06	0.24**	–0.21**	–0.18*	–0.04
Bedroom temperature	0.13*	0.13*	–0.20*	0.04	0.16*
Outdoor temperature	0.15**	0.18**	–0.28*	–0.09	0.11
*0.05 < p < 0.1. **p < 0.05.							

## Discussion

Many children with asthma have underlying allergies (Burrows et al. 1989; Remes and Korppi 1996; von Mutius et al. 1994), and for these children, indoor environmental allergens can exacerbate their symptoms. This is especially true in water-damaged areas such as post-Katrina NOLA, where concentrations of mold and other indoor allergens, including those that increase in moist environments such as dust mite, cockroach, and endotoxin, can reach high levels. In the months after Katrina, the concentrations of airborne mold, glucan, and endotoxin (Chew et al. 2006; Rao et al. 2007; Solomon et al. 2006) were of concern because they had reached levels previously associated with respiratory pathology, wheeze, and asthma in other studies (Douwes et al. 2002; Reynolds et al. 2001; Rockwell 2005; Rylander et al. 1998).

A major goal of HEAL was to characterize the post-Katrina environmental exposures of children with asthma. Therefore, we evaluated HEAL children’s homes for mold, allergens, and other asthma triggers at study baseline. Airborne mold levels vary over short periods of time, and cut-offs for normal mold exposure levels and levels associated with symptoms are not well established (Horner et al. 2008). Instead, characterization of potential mold exposure problems in a home is determined by the type of mold present (e.g., water-associated molds and soil mold spores rarely dominate in houses free of visible mold or water damage) and the ratio of indoor to outdoor mold (e.g., indoor is expected to be lower than outdoor levels) (Horner et al. 2008). When looking at the types of spores detected from HEAL samples, mold found indoors reflected what was detected outdoors; molds associated with water damage (e.g., *Stachybotrys, Chaetomium, Ulocladium*) were uncommon (3–18% of homes) and the most common molds were leaf surface/outdoor molds (e.g., Ascospores, *Cladosporium,* Basidiospores in 92–98% of homes). We found the average levels of indoor airborne mold to be lower than outdoor airborne mold, even for HEAL homes that had been flooded (geometric means of 627 indoor/4,954 outdoor spores/m^3^), in contrast with measurements taken within 2 months (11,000–645,000 indoor/21,000–102,000 outdoor spores/m^3^) (Solomon et al. 2006) and 1 month (356,000 indoor/43,000 outdoor spores/m^3^) (Rao et al. 2007) after Katrina.

These levels detected in HEAL compared with the other post-Katrina studies might not only reflect a difference in when the samples were collected and the state of the homes they were collected from, but may also reflect sampling time variability [10 min vs. 36–144 min (Rao et al. 2007) and 6–24 hr (Solomon et al. 2006)]. Other limitations in HEAL air sampling were the limited number of samples collected (i.e., two from each room), sample collection on a single day at a single point in time, and use of the Air-O-Cell/light microscopy method for mold identification. Previous research suggests that use of the Air-O-Cell/light microscopy method in combination with culturable mold identification, such as that used with the Andersen sampler, might provide a more representative indication of indoor mold levels and types (Heinsohn 2007; Tsai et al. 1999). The levels found in HEAL might also be a result, in part, of a natural reduction of mold and allergens as flood waters receded and debris piles were removed. They might also have been attributable to extensive home renovation and mold remediation measures conducted by NOLA residents after the storm. Renovation and mold remediation can be effective at reducing mold. In a demonstration project conducted shortly after Katrina, marked reductions in indoor airborne mold levels were observed in postrenovation homes compared with prerenovation homes, sometimes of multiple orders of magnitude (Chew et al. 2006). Similar reductions were observed in another study of 17 homes in the greater Kansas City, Missouri, area that underwent renovation/remediation because of visible mold and health concerns (Barnes et al. 2007). At HEAL baseline, a significant portion of HEAL children (*n* = 68) were living in homes that had flooded post-Katrina. Of these, 41 homes had completed renovation or mold remediation before HEAL baseline. Unfortunately, only 22 of the 41 caregivers in these homes were able to provide us with detailed information about the specific measures taken (Table 1), which was not enough data for a detailed analysis.

Another finding in HEAL was that flooded homes, regardless of renovation and remediation, had higher levels of indoor airborne mold than nonflooded homes. Although this finding was of borderline statistical significance (*p =* 0.07), the pattern persisted across several subsets of the study population. For example, airborne mold levels were higher in single-family homes that had flooded than in nonflooded single-family homes (*p =* 0.05) and when the child lived in the same home during Katrina that he or she lived in at HEAL baseline (*p =* 0.02). Living in a different location (instead of the same location) may be an indication that the home was new and therefore clean; however, we did not ask how old the home was. Homes that were both flooded and rain-damaged had some of the highest mold levels observed in the study (742 spores/m^3^), and these levels were significantly higher than in homes that sustained no water damage by flooding or a roof leak (438 spores/m^3^, *p =* 0.05; *p*-value not shown in Table 4). It would be informative to monitor indoor mold levels in these and other water-damaged homes over longer periods of time and to conduct a detailed analysis to examine relationships between mold levels and specific remediation measures.

Another interesting finding in HEAL was that although indoor airborne mold levels were lower than outdoor levels and substantially lower than what had been found within 2 months after Hurricane Katrina, *Alternaria* antigen was prevalent in dust samples collected from the children’s bedrooms; it was detected in almost all (98%) bedrooms and in 58% of bedrooms at concentrations > 10 µg/g. This relationship was unexpected and requires further investigation. *Alternaria* is a common fungus, and at high exposure levels, it has been associated with the onset of childhood asthma and with severe asthma reactions in sensitive individuals (Bush and Prochnau 2004; Downs et al. 2001; Lemanske 2002; Salo et al. 2005). *Alternaria*, like most molds, grows in humid areas and has been found in water-damaged homes, especially those that have cellulose in their building materials (Ren et al. 1998) (e.g., wood, insulation, ceiling tiles). Whether *Alternaria* was a significant asthma trigger in HEAL will require further investigation. It is possible that the high prevalence and concentration of *Alternaria* in HEAL bedroom dust samples were artifacts of the assay technique. Other fungi, such as those belonging to the *Pleosporaceae* family (*Alternaria, Ulocladium, Stemphylium*) and other dematiaceous genera including *Epicoccum,* can cross-react with the polyclonal antibody used in the *Alternaria* inhibition ELISA assay and artificially increase the results (Schmechel et al. 2008); these fungi were detected in HEAL air samples. Polymerase chain reaction analyses of HEAL dust (Vesper et al. 2007a, 2007b) will provide us with a more comprehensive mold profile. These results will be important in relating mold species to asthma morbidity as comparative genomic studies have indicated that certain allergens (Alt a 1, for example) may be shared between different species of fungi (Bowyer et al. 2006; Saenz-de-Santamaria et al. 2006).

Compared with *Alternaria*, other allergens (Bla g 1, Der p 1, and Mus m 1) were less abundant in dust samples (Table 2). Another post-Katrina study conducted at about the same time as HEAL also found low dust mite allergen levels; Der p 1 was detected from 14.7% of homes (1.6–18.7 μg/g from 3 of 7 occupied flooded homes and 2 of 27 unoccupied flooded homes) (Adhikari et al. 2010), compared with 35% of homes with detectable Der p 1 in HEAL (0.20–18.5 μg/g from 181 homes). This is in contrast with concentrations reported by the New Orleans Healthy Home Initiative (HHI), which was conducted several months before Katrina (Rabito et al. 2007), where Der p 1 exceeded 2 μg/g in 63.9% of homes compared with 9% of HEAL homes exceeding this level. Cockroach allergens detected in dust samples were also higher in HHI than in HEAL (Bla g 1 > 2U/g in 90.3% of homes in HHI vs. 4% in HEAL) (Rabito et al. 2007). The low concentrations in HEAL bedrooms might have been attributable partly to renovations and mold remediation or to children moving to new, cleaner homes. Environmental sampling techniques also might have contributed to the differences in allergen concentrations. In HEAL, dust from the children’s beds and bedroom floors was analyzed, whereas in HHI, dust was collected from the living room floor and upholstery, child’s bedroom floor and bed, and kitchen floor, and the highest value for the home from any of these locations was reported.

Similar to airborne concentrations of mold, geometric mean levels of dust-borne endotoxin and glucan in HEAL homes were low compared with other environmental studies (11.4 EU/mg in HEAL vs. 20 and 44 EU/mg endotoxin) (Park et al. 2000; Thorne et al. 2009), (0.5 μg/g in HEAL vs. 2,743 μg/g (1□3)-β-glucan and 55.1 μg/g (1□3)-β-d-glucan) (Bertelsen et al. 2010; Iossifova et al. 2007). However, the concentrations of endotoxin in HEAL are still of concern because they have previously been associated with increased asthma symptoms and use of asthma medications (Rizzo et al. 1997).

When we tested for correlations between mold, indoor allergens, and other home environment conditions, we found that the negative correlation between indoor airborne mold and dust *Alternaria* antigen concentrations described above was significant (*p =* 0.04). This result was unexpected and requires further investigation. Other correlations were as expected, such as the correlations between dust glucan and *Alternaria* antigen concentrations and between indoor airborne mold levels and bedroom relative humidity.

## Conclusion

Before HEAL, flood waters had receded, most of the debris piles had been removed, and mold levels were decreasing naturally on their own. In addition, steps taken through public awareness programs to ensure that no one was living in mold-ridden homes seemed to be effective because no HEAL children were found living in unhealthy conditions from copious amounts of mold growing throughout a room or home. Although we found only low-to-moderate concentrations of airborne mold and dust allergens, the levels of certain exposures (endotoxin, *Alternaria* antigen) had previously been associated with increased asthma morbidity.

## Supplemental Material

(135 KB) PDF
