# Supplementary material for: Indoor Environmental Exposures for Children with Asthma Enrolled in the HEAL Study, Post-Katrina New Orleans
Source: Environ Health Perspect. 2012 Aug 15;120(11):1600–6. doi: 10.1289/ehp.1104840 (PMC3556612; doi:10.1289/ehp.1104840)
Supplement: (135 KB) PDF [file ehp.1104840.s001.pdf]

Supplemental Material

Indoor Environmental Exposures for Children with Asthma Enrolled in the HEAL Study, Post-Katrina New Orleans

L. Faye Grimsley, Patricia C. Chulada, Suzanne Kennedy, LuAnn White, Jeremy Wildfire, Richard D. Cohn, Herman Mitchell, Eleanor Thornton, Jane El-Dahr, Mosanda M. Mvula, Yvonne Sterling, William J. Martin, Kevin U. Stephens, Maureen Lichtveld

Contents

Supplemental Material, Figure S1. Baseline home environmental evaluations took place over a 17-month period, from March 2007 through August 2008. The lines (smoothed plot) represent all indoor and outdoor airborne mold levels (spores/m^3) measured during the baseline home environmental evaluations. The shaded areas around the lines depict the 95% confidence intervals. 2

Supplemental Material, Table S1: Concentrations of airborne mold in Flooded HEAL homes at baseline (N=68 homes) 3

Supplemental Material, Table S2: Concentrations of airborne mold in Non-Flooded HEAL homes at baseline (N=114 homes) 4

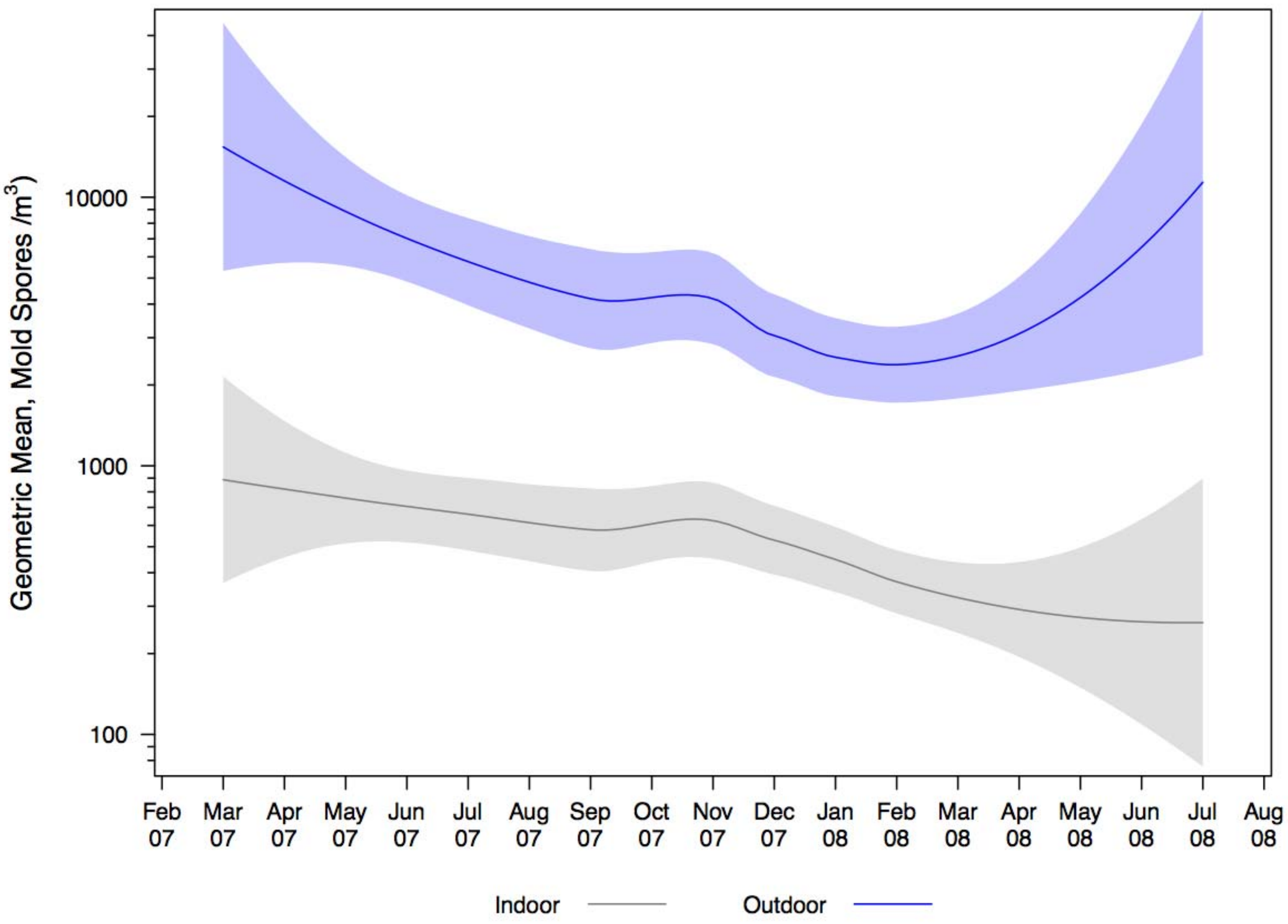

Supplemental Material, Figure S1. Baseline home environmental evaluations took place over a 17-month period, from March 2007 through August 2008. The lines (smoothed plot) represent all indoor and outdoor airborne mold levels (spores/m^3) measured during the baseline home environmental evaluations. The shaded areas around the lines depict the 95% confidence intervals.

Supplemental Material, Table S1: Concentrations of airborne mold in Flooded HEAL homes at baseline (N=68 homes)

	Indoor Molds			Outdoor Molds		
Rank	Mold Type	N (%) Detectable	Geometric Mean (GSE)	Mold Type	N (%) Detectable	Geometric Mean (GSE)
1st	Basidiospores	68 (100%)	225 (45.0)	Basidiospores	68 (100%)	2055 (483.6)
2nd	*Cladosporium*	66 (97%)	67 (13.0)	*Cladosporium*	67 (99%)	435 (91.2)
3rd	Ascospores	65 (97%)	43 (7.4)	Ascospores	68 (100%)	323 (65.8)
4th	*Penicillium-Aspergillus*	56 (82%)	20 (4.5)	*Penicillium-Aspergillus*	53 (78%)	22 (5.3)
5th	*Curvularia*	55 (81%)	14 (2.6)	*Ganoderma*	52 (76%)	18 (4.3)
6th	Unidentified	47 (69%)	7 (1.1)	*Curvularia*	47 (69%)	11 (2.7)
7th	*Bipolaris*	39 (57%)	5 (0.9)	*Cercospora*	47 (69%)	9 (1.9)
8th	*Pithomyces*	58 (66%)	5 (0.8)	*Alternaria*	43 (63%)	7 (1.5)
9th	*Alternaria*	29 (43%)	3 (0.5)	Unidentified	42 (63%)	7 (1.7)
10th	*Ganoderma*	26 (38%)	3 (0.4)	*Bipolaris*	37 (54%)	6 (1.2)
11th	*Epicoccum*	19 (28%)	2 (0.3)	*Epicoccum*	34 (50%)	3 (0.6)
12th	*Chaetomium*	15 (22%)	2 (0.2)	*Pithomyces*	28 (41%)	3 (0.4)
13th	*Arthrinium*	10 (15%)	2 (0.2)	*Pestalotia*	15 (22%)	2 (0.2)
14 th	*Ulocladium*	11 (16%)	1 (0.2)	*Chaetomium*	10 (15%)	1(0.2)
15th	*Cercospora*	10 (15%)	1 (0.1)	*Arthrinium*	5 (7%)	1(0.1)
16th	*Pestalotia*	6 (9%)	1 (0.1)	*Stemphylium*	5 (7%)	1(0.1)
17th	*Stemphylium*	3 (4%)	1 (0.1)	*Stachybotrys*	3 (4%)	1(0.1)
18th	*Stachybotrys*	3 (4%)	1 (0.1)	*Ulocladium*	2 (3%)	1(0.1)
19th	*Trichoderma*	0 (0%)	0 (0)	Rust	2 (3%)	1(0.1)
20th	Rust	0 (0%)	0 (0)	*Trichoderma*	1 (1%)	1(0.1)
21st	Other	0 (0%)	0 (0)	Other	0 (0%)	0 (0)

Supplemental Material, Table S2: Concentrations of airborne mold in Non-Flooded HEAL homes at baseline (N=114 homes)

	Indoor Molds			Outdoor Molds		
Rank	Mold Type	N (%) Detectable	Geometric Mean (GSE)	Mold Type	N (%) Detectable	Geometric Mean (GSE)
1st	Basidiospores	111 (97%)	110 (16.9)	Basidiospores	113 (99%)	1002 (200.1)
2nd	*Cladosporium*	110 (96%)	63 (9.2)	*Cladosporium*	113 (99%)	510 (80.2)
3rd	Ascospores	102 (89%)	27 (3.9)	Ascospores	114 (100%)	215 (30.5)
4th	*Penicillium-Aspergillus*	86 (75%)	16 (3.1)	*Penicillium-Aspergillus*	80 (70%)	17 (3.4)
5th	*Curvularia*	85 (75%)	12 (2.1)	*Ganoderma*	82 (72%)	13 (2.3)
6th	*Bipolaris*	67 (59%)	5 (0.7)	*Curvularia*	75 (66%)	11 (2.2)
7th	Unidentified	64 (56%)	5 (0.7)	Unidentified	75 (66%)	9 (1.6)
8th	*Pithomyces*	62 (54%)	5 (0.9)	*Cercospora*	47 (59%)	6 (1.0)
9th	*Alternaria*	43 (37%)	2 (0.3)	*Alternaria*	75 (66%)	7 (1.0)
10th	*Ganoderma*	34 (30%)	2 (0.2)	*Bipolaris*	71 (62%)	5 (0.8)
11th	*Epicoccum*	32 (28%)	2 (0.2)	*Epicoccum*	48 (42%)	3 (0.4)
12th	*Ulocladium*	21 (18%)	2 (0.2)	*Pithomyces*	39 (34%)	3 (0.4)
13th	*Pestalotia*	14 (12%)	1 (0.1)	*Pestalotia*	27 (24%)	2 (0.1)
14th	*Cercospora*	13 (11%)	1 (0.1)	*Arthrinium*	8 (7%)	1 (0.1)
15th	*Chaetomium*	12 (11%)	1 (0.1)	*Chaetomium*	7 (6%)	1 (0.1)
16th	*Arthrinium*	7 (6%)	1 (0.1)	*Ulocladium*	7 (6%)	1 (0.1)
17th	*Stemphylium*	5 (4%)	1 (0.1)	*Stachybotrys*	5 (4%)	1 (0.1)
18th	*Stachybotrys*	3 (3%)	1 (0.04)	*Stemphylium*	3 (3%)	1 (0.04)
19th	Rust	2 (2%)	1 (0.02)	Rust	3 (3%)	1 (0.03)
20th	*Trichoderma*	1 (1%)	1 (0.1)	*Trichoderma*	0 (0%)	0 (0)
21st	Other	0 (0%)	0 (0)	Other	0 (0%)	0 (0)
